# Effect of CaO on Phase Composition and Properties of Aluminates for Barium Tungsten Cathode

**DOI:** 10.3390/ma11081380

**Published:** 2018-08-08

**Authors:** Jinglin Li, Jianjun Wei, Yongbao Feng, Xiaoyun Li

**Affiliations:** 1 College of Materials Science and Engineering, Nanjing Tech University, Nanjing 210009, China; 662080502016@njtech.edu.cn (J.L.); moruoxin@njtech.edu.cn (J.W.); lixiaoyun@njtech.edu.cn (X.L.); 2 Jiangsu Collaborative Innovation Center for Advanced Inorganic Function Composites, Nanjing 210009, China

**Keywords:** aluminates, molar amount of CaO, phase composition, emission current, evaporation rate

## Abstract

6BaO·xCaO·2Al_2_O_3_ (x = 0.8, 1.2, 1.6, 2, and 2.2) aluminates were synthesized via a liquid phase co-precipitation method. Effects of the molar amount of CaO on the phase of aluminates before and after melting and their hygroscopic phase, melting properties, environmental stability, evaporation, and emission properties were systematically studied. The results show that with the increase of the molar amount of CaO, the aluminates change from a mixture phase to a single phase of Ba_3_CaAl_2_O_7_, and the diffraction peak shifts to a higher angle. The melted phase of the aluminates changed from a single phase to a mixed phase of Ba_5_CaAl_4_O_12_ and Ba_3_CaAl_2_O_7_. Meanwhile, the comprehensive properties of the aluminates are improved. The weight gain of 6BaO·2CaO·2Al_2_O_3_ aluminates is only 10.88% after exposure to air for 48 h; the pulse emission current density of barium tungsten cathodes impregnated with 6BaO·2CaO·2Al_2_O_3_ aluminates in the porous tungsten matrix can reach 28.60 A/cm^2^ at 1050 °C, and the evaporation rate is 2.52 × 10^−10^ g/(cm^2^·s).

## 1. Introduction

As the main electron source for vacuum electronic devices, the impregnated barium-tungsten (Ba-W) cathode has the desirable characteristics of high emission current density, long lifetime, resistance to ion bombardment, and so on [1,2]. It is prepared by impregnating a molten electron emission salt into the porous tungsten matrix, followed by processing and coating with a film [3]. The composition of the electron emission source has an important effect on the emission performance and lifetime of the Ba-W cathode [4,5]. The most widely used electronic emission materials in Ba-W cathodes are aluminates. According to the different molar ratios of BaO, CaO and Al_2_O_3_ in aluminates, they can be categorized as 612 (i.e., n_BaO_:n_CaO_:n_Al2O3_ = 6:1:2) [6,7], 411 [8], 532 [9], and 311 [10] aluminates.

To improve the properties of aluminates for Ba-W cathodes, researchers have focused on the effects of the synthesis process [8,11,12], the phase composition [6,13], the scandium element doping [14,15,16], and porous tungsten matrix [17] on the properties of aluminates. C. Lai et al. [14,18,19] proposed the sputtering of a layer of unit or multi-element metal film on the surface of Ba-W cathode to prepare a coated cathode, which can improve its emission performance. However, there are fewer studies on the relationship between chemical composition, structure, and properties of aluminates. Ba and BaO are used as sources of electron emission in aluminates, and their molar amount has a direct effect on emission and evaporation of the cathode [20,21]. CaO cannot provide emission electrons, but it has an important influence on the environmental stability, melting characteristics, and phase composition of aluminates, which has an indirect effect on the emission and evaporation properties of aluminates for Ba-W cathodes [22]. 6BaO·CaO·2Al_2_O_3_ is used widely in electron emitting aluminates, but the effect of CaO content on its structure and properties has rarely been reported.

In this paper, 6BaO·xCaO·2Al_2_O_3_ (x = 0.8, 1.2, 1.6, 2, and 2.2) aluminates were prepared by a liquid phase method. Effects of the molar amount of CaO on the phase composition, melting characteristics, environmental stability of aluminates, and the performance of Ba-W cathodes were systematically studied.

## 2. Materials and Methods

### 2.1. Experimental Process

In this paper, analytical purities of Ba(NO_3_)_2_ (≥99.5%, Guo Yao Group Co., Ltd., Shanghai, China), Ca(NO_3_)_2_·4H_2_O (≥99.0%, Guo Yao Group Co., Ltd., Shanghai, China), and Al(NO_3_)_3_·9H_2_O (≥99.0%, Guo Yao Group Co., Ltd., Shanghai, China) were used as raw materials. The three nitrates were weighed at a molar ratio and formulated into a mixed salt solution and then placed in a water bath at 10 °C. A mixture of (NH_4_)_2_CO_3_ (≥99.0%, Ling Feng Chemical Reagent Co., Ltd., Shanghai, China) and NH_3_·H_2_O (Ling Feng Chemical Reagent Co., Ltd., Shanghai, China) [23] (W((NH_4_)_2_CO_3_) = 14.09%, W(25–28% NH_3_·H_2_O) = 6.09%) was then rapidly added to the mixed salt solution while stirring until the pH = 8.5 [6]. Five kinds of 6BaO·xCaO·2Al_2_O_3_ aluminate precursors were obtained after the reaction products were aged, washed by centrifugation, and dried.

Each of the five precursor powders were compacted into Φ 15 mm × 30 mm cylindrical samples by cold isostatic pressing technology at 300 MPa for 90 s. The samples were placed in a high-purity alumina crucible and calcined in an air atmosphere at 1500 °C for 2 h, followed by a cooling rate of 2 °C per minute. The 6BaO·xCaO·2Al_2_O_3_ aluminate powders were obtained by grinding sintered samples in an agate mortar, which were then vacuum-packed.

### 2.2. Performance Testing

Phase identification of aluminates before and after melting and their hygroscopic phase were determined via X-ray powder diffractometer (XRD) (Rigaku, SmartLab, Tokyo, Japan) using Cu-Kα radiation and step scanning over a 2θ range from 10° to 90°, with a scan rate of 10°/min. Melting characteristics of aluminates pressed into a cylinder of Φ 8.5 mm × 7 mm were investigated by a high temperature real-time observation and test system (TOM-AC, CeDeD equipment R&D center, Dresden, Germany). The environmental stability of the aluminates at 25 °C and 50% RH were measured while using a constant temperature and humidity chamber (LHS-100HC, Shanghai Aozhen Instrument Manufacturing Co., Ltd., Shanghai, China).

The aluminates were impregnated into a tungsten matrix with a porosity of 25–26%. After processing and film mulching, an impregnated Ba-W cathode of Φ 3.4 mm × 1 mm was prepared. The evaporation rate of aluminates at 1050–1200 °C were measured by the Baker method using an electronic injection analysis system (AD-NTECH, Yanhua Science and Technology Co., Ltd., Nanjing, China). The impregnated cathode was assembled into a water-cooled anode diode, and its pulse emission performance at 1050 °C was tested after activation at 1200 °C, using the cathode performance test system (YYC-10, Yanhua Technology Co., Ltd., Yangzhou, China), which the accuracy of ammeter is 0.01 A.

## 3. Results

### 3.1. Phase Composition of 6BaO·xCaO·2Al_2_O_3_ Aluminates

**Figure 1:**
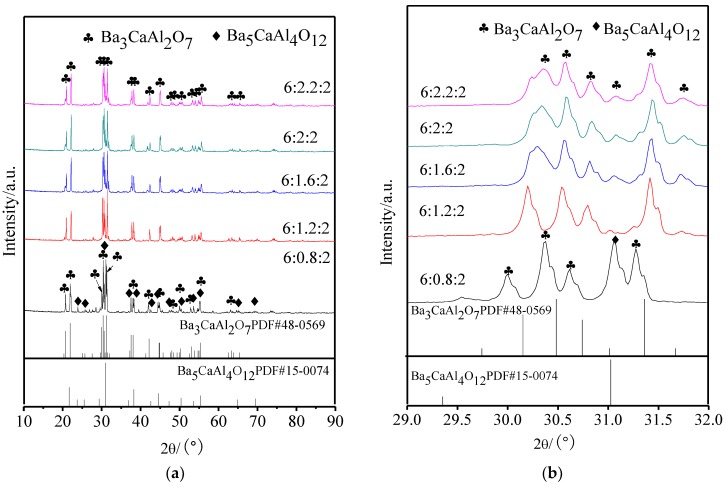
X-ray powder diffractometer (XRD) patterns of 6BaO·xCaO·2Al_2_O_3_ aluminates. (**a**) XRD patterns in 10~90°; and, (**b**) Partial enlarged drawing in 29~32° of (**a**).

Figure 1 displays the XRD patterns of aluminates with different molar amounts of CaO; Figure 1b is a partial enlarged drawing of 2θ in the range of 29° to 32°. It could be seen from Figure 1a that the aluminate phase was a mixture of Ba_3_CaAl_2_O_7_ and Ba_5_CaAl_4_O_12_ when the molar amount of CaO was 0.8, and when the molar amount of CaO was more than 1.2, only a single Ba_3_CaAl_2_O_7_ phase was seen. From Figure 1b, it could be determined that, with the increase of the molar amount of CaO, the diffraction peak of Ba_3_CaAl_2_O_7_ at 30–31° became wider and shifts to a higher angle.

When x decreased from 2 to 1.2, the main crystal phases of 6BaO·xCaO·2Al_2_O_3_ were all Ba_3_CaAl_2_O_7_ phase, and the corresponding decrease of Ca^2+^ did not cause any change of the main crystal phase structure of aluminates. According to the diffraction peak shifts to lower angles, the absence of Ca^2+^ only caused lattice distortion, and the corresponding interplanar spacing increased. When the molar amount of CaO was 0.8, the structure of Ba_3_CaAl_2_O_7_ phase was unstable due to the many Ca^2+^ vacancies, and the second phase of Ba_5_CaAl_4_O_12_ was formed. When the molar amount of CaO increased from 2 to 2.2, the excess CaO had no effect on the phase composition of aluminates, and the diffraction peak continued to shift to a higher angle, which suggested that Ca^2+^ might be filling in the lattice of the Ba_3_CaAl_2_O_7_ phase in the form of interstitial ions, so that the interplanar spacing decreased [24]. The reason for the broadening of the diffraction peak might be that the increase of CaO made the crystallization degree of 6BaO·xCaO·2Al_2_O_3_ aluminates lower, and the grain size decreased.

### 3.2. Melting Characteristic of 6BaO·xCaO·2Al_2_O_3_ Aluminates

**Figure 2:**
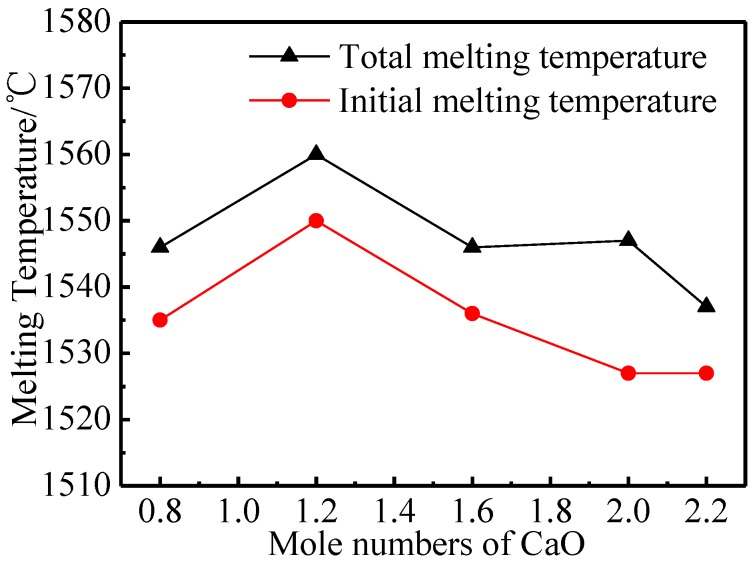
Melting properties of 6BaO·xCaO·2Al_2_O_3_ aluminates.

Melting characteristics can determine the impregnation effect of aluminates—under the same impregnation conditions, aluminates with lower melting temperatures will have a higher impregnation rate. Figure 2 displays the melting characteristics of five kinds of aluminates. The initial melting temperature and the total melting temperature of the aluminates increased first and then decreased with the increase of the molar amount of CaO.

When the molar amount of CaO increased from 0.8 to 1.2, the initial melting and total melting temperature gradually increased [25], so that the 6BaO·1.2CaO·2Al_2_O_3_ could only be completely melted at 1560 °C. This was perhaps because the phase of aluminates was gradually transformed from the mixed phase of Ba_3_CaAl_2_O_7_ and Ba_5_CaAl_4_O_12_ to the single phase of Ba_3_CaAl_2_O_7_ as the molar amount of CaO increased. The mixed phase might be slightly eutectic, which led to lower initial melting and total melting temperatures, while the melting temperature of the single phase Ba_3_CaAl_2_O_7_ was higher. Then, the melting temperature decreased with the increase of the molar amount of CaO, which might be because the phase of aluminates was still dominated by the Ba_3_CaAl_2_O_7_ phase when the molar amount of CaO was greater than 1.2. There were more defects in the crystals and the grain size decreases, thus lowering the melting temperature.

### 3.3. Molten Phase of 6BaO·xCaO·2Al_2_O_3_ Aluminates

**Figure 3:**
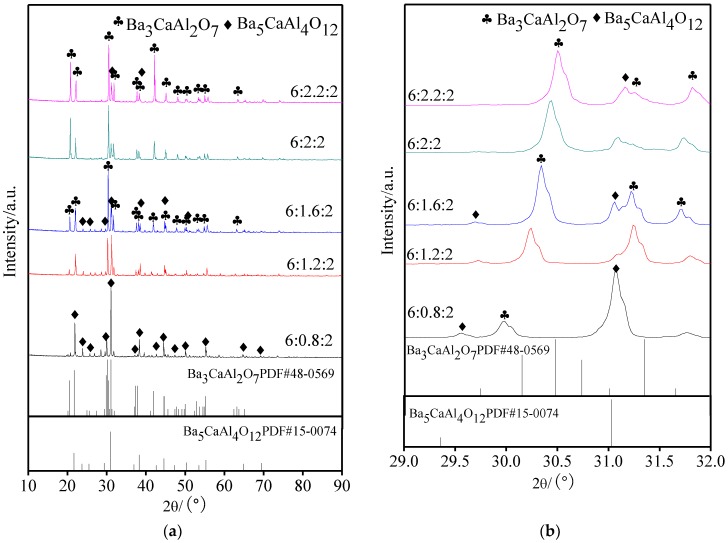
XRD patterns of molten 6BaO·xCaO·2Al_2_O_3_ aluminates; (**a**) XRD patterns in 10~90°; and, (**b**) Partial enlarged drawing in 29~32° of (**a**).

It is very important to analyze the molten phase of aluminates, which reacts with the tungsten matrix to form active substances, for the study of factors that are affecting the cathode properties. Five kinds of aluminates were rapidly heated to 1690 °C and then cooled after 90 s. XRD patterns of molten aluminates with different molar amounts of CaO are shown in Figure 3, with Figure 3b displaying a partial enlarged drawing of 2θ in the range of 29° to 32°. The structure and properties of five kinds of aluminates with different molar amounts of CaO were displayed in Table 1.

Combining Figure 3 and Table 1, the phase composition of aluminates before and after melting was changed. When the molar amount of CaO was 2.0 or 2.2, the molten phases of aluminates were Ba_3_CaAl_2_O_7_ with a very small amount of Ba_5_CaAl_4_O_12_, and the fused Ba_3_CaAl_2_O_7_ phase exhibited the preferential growth of individual crystal planes. When the molar amount of CaO was less than or equal to 1.6, the phase changed from Ba_3_CaAl_2_O_7_ to the coexistence of Ba_3_CaAl_2_O_7_ and Ba_5_CaAl_4_O_12_, and the content of the Ba_5_CaAl_4_O_12_ phase was increasing. This indicated that the aluminates of the Ba_3_CaAl_2_O_7_ phase, which were unstably synthesized with different CaO proportions, led to the deficiency or excess of Ca^2+^ due to the vacancies or the interstitials of CaO, so the phase separation would occur in the process of high temperature melting and cooling.

### 3.4. Environmental Stability of 6BaO·xCaO·2Al_2_O_3_ Aluminates

**Figure 4:**
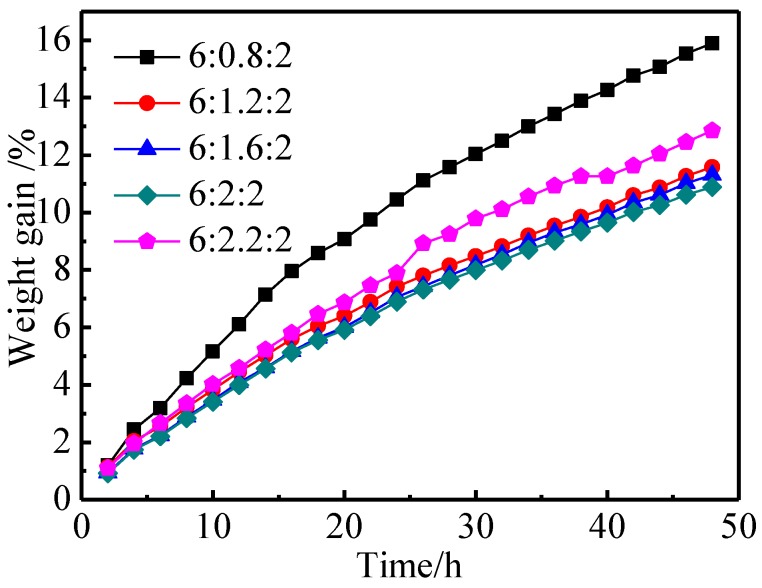
Weight gain of 6BaO·xCaO·2Al_2_O_3_ aluminates exposed to the air for different time.

The aluminates exposed to air can react with CO_2_, water vapor, and so on, and the macroscopic evidence for this is the increase in mass. The degree of moisture absorption and CO_2_ absorption of aluminates can be judged from their weight gain rate [26]. Aluminates with low weight gain and high environmental stability will ensure the consistency and stability of the products in the cathode production process. Figure 4 shows the weight gain curves of aluminates with different molar amounts of CaO over 48 h. As illustrated in Figure 4, when the molar amount of CaO in aluminates was increased from 0.8 to 2.0, the weight gain rate of aluminates gradually decreased from 15.89% to 10.88% for 48 h, and the environmental stability gradually improved [5], which might be related to the perfection of crystal structure and the formation of single-phase Ba_3_CaAl_2_O_7_. When the molar amount of CaO was 2.2, the weight gain rate of aluminates increased, and the environmental stability became worse, which might be related to the Ca^2+^ entering into the interstitial position of Ba_3_CaAl_2_O_7_ and the structure becoming too loose. From another point of view, it could be deduced that the aluminates of single phase Ba_3_CaAl_2_O_7_ had high environmental stability. At the same time, the slope of the weight gain curve decreased gradually with the prolongation of storage time, which meant that the ability of the aluminates to absorb moisture and CO_2_ gradually became saturated. For 6BaO·xCaO·2Al_2_O_3_ aluminates, the proper amount of CaO could increase the stability of aluminates to a certain extent.

**Figure 5:**
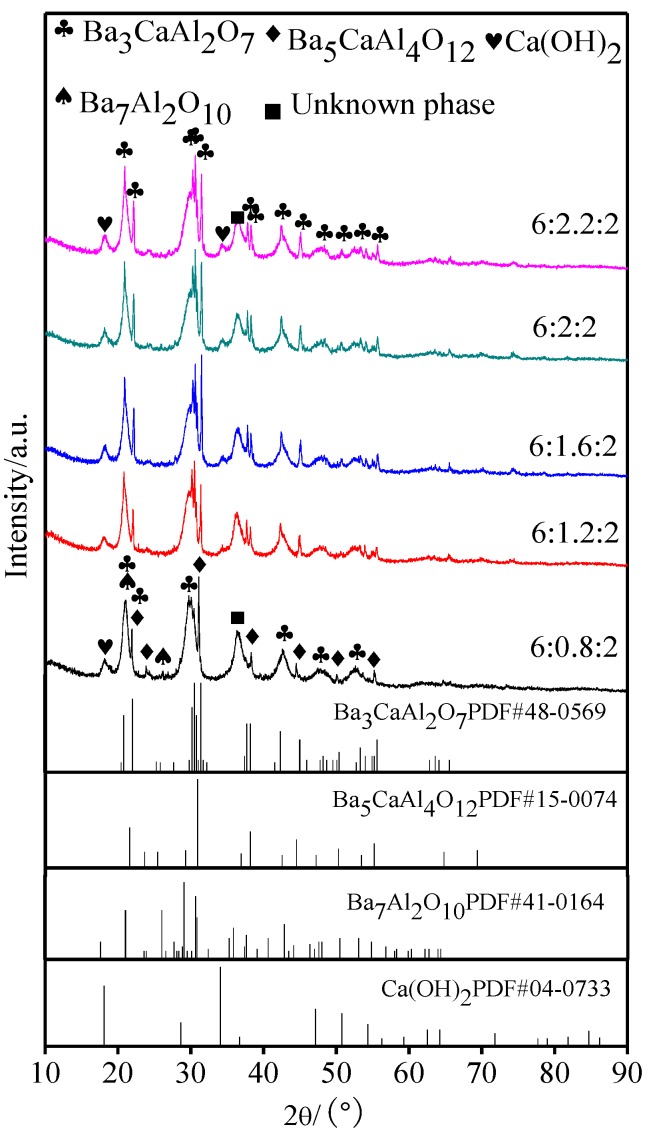
XRD patterns of 6BaO·xCaO·2Al_2_O_3_ aluminates exposured to the air for 48 h.

The XRD patterns of the aluminates that were exposed to air for 48 h are shown in Figure 5. When compared with Figure 1 and Figure 5, it could be seen that the diffraction peaks of aluminates appeared to broaden after moisture absorption and weight gain, and the reaction of aluminates with H_2_O and CO_2_ in air resulted in the gradual disappearance of Ba_3_CaAl_2_O_7_ phase structure and the formation of new phases, which included Ca(OH)_2_, Ba_7_Al_2_O_10_, and other unknown phases [26,27]. Three characteristic diffraction peaks of Ba_3_CaAl_2_O_7_ phase disappeared at 29~30° due to the serious hygroscopic absorption of aluminates with a molar ratio of 6:0.8:2.

### 3.5. Emission Performance of 6BaO·xCaO·2Al_2_O_3_ Aluminates

**Figure 6:**
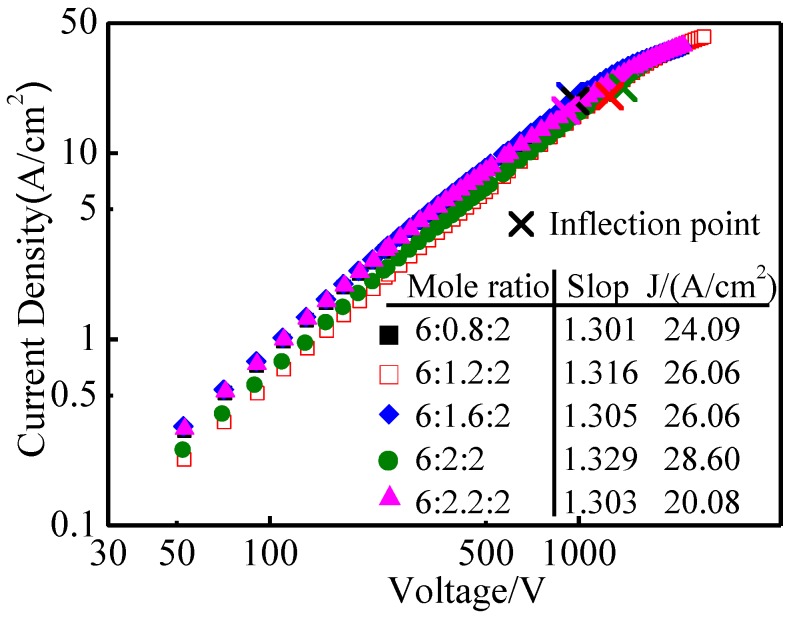
Pulse emission curves of 6BaO·xCaO·2Al_2_O_3_ aluminates barium tungsten cathodes.

The pulse emission curves of aluminate-Ba-W cathodes with different molar amounts of CaO are shown in Figure 6. It could be seen from the diagram that the pulse emission current densities of five kinds of aluminate-Ba-W cathode with CaO molar amounts of 0.8, 1.2, 1.6, 2, and 2.2 at 1050 °C were 24.09 A/cm^2^, 26.06 A/cm^2^, 26.06 A/cm^2^, 28.60 A/cm^2^, and 20.08 A/cm^2^, respectively. When the molar amount of CaO increased, the pulse emission current density of aluminates increased first and then decreased; among them, 6BaO·2CaO·2Al_2_O_3_ aluminate was the highest.

Combined with the molten phase results of 6BaO·xCaO·2Al_2_O_3_ aluminates, it could be seen that as the molar amount of CaO increased from 0.8 to 2.0, the content of BaO decreased gradually, but its emission property was improved, which indicated that the molten phase of aluminates played a decisive role in its emission performance. There was a very small amount of Ba_5_CaAl_4_O_12_ in the molten phase of 6BaO·2CaO·2Al_2_O_3_ aluminates, while the main crystalline phase was Ba_3_CaAl_2_O_7_, but the second phase was present in higher amounts in other aluminates with different molar amounts of CaO [28]. Thus, the Ba_3_CaAl_2_O_7_ phase might have higher reactivity with W [6], producing more Ba and BaO, leading to the Ba-W cathode having a higher emission current density. However, the emission property of aluminates decreased when the molar amount of CaO was 2.2, which might be due to the decrease of BaO content in the aluminates and the presence of more of the non-reactive second phase in the molten product. Therefore, the reaction activity of the melt product and tungsten matrix decreased, which led to the decrease of the emission property.

### 3.6. Evaporation Rate of 6BaO·xCaO·2Al_2_O_3_ Aluminates

**Figure 7:**
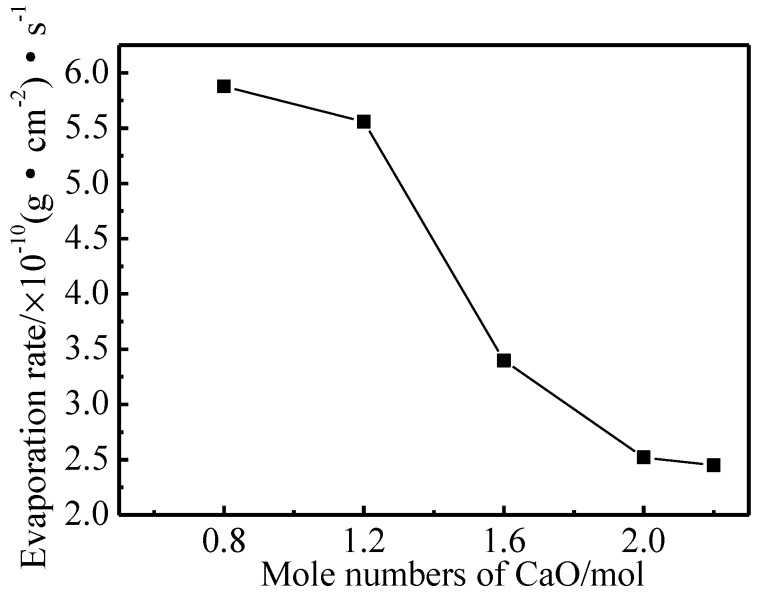
Average evaporation rate diagram of cathodes filled with 6BaO·xCaO·2Al_2_O_3_ aluminates.

The evaporation rate curve of Ba-W cathodes that were prepared with five kinds of aluminates at 1050 °C are shown in Figure 7. It could be clearly seen that the average evaporation rate of impregnated aluminate-Ba-W cathodes decreased with the increase of the molar amount of CaO [25], which indicated that this increase could reduce the evaporation of electronically active substances and prolong the lifetime of the cathode at working temperatures. The evaporation rate of barium tungsten cathodes that were impregnated with 6BaO·2CaO·2Al_2_O_3_ aluminates in the porous tungsten matrix was only 2.52 × 10^−10^ g/(cm^2^·s). The mass ratio of Ba and BaO, as active vaporizers, determined the evaporation rate of the aluminates and usually P_Ba_/P_BaO_ > 1 [29,30], so the main evaporative specie of the Ba-W cathode was Ba. The molten phase of aluminates tended towards a single phase of Ba_3_CaAl_2_O_7_ with the increase of the molar amount of CaO, which indicated that the Ba_3_CaAl_2_O_7_ phase aluminates were likely to produce a BaO emitter with a lower evaporation rate when compared with the Ba_5_CaAl_4_O_12_ phase aluminates, while Ba_5_CaAl_4_O_12_ phase aluminates easily reacted with W to produce a high evaporation rate of Ba.

**Figure 8:**
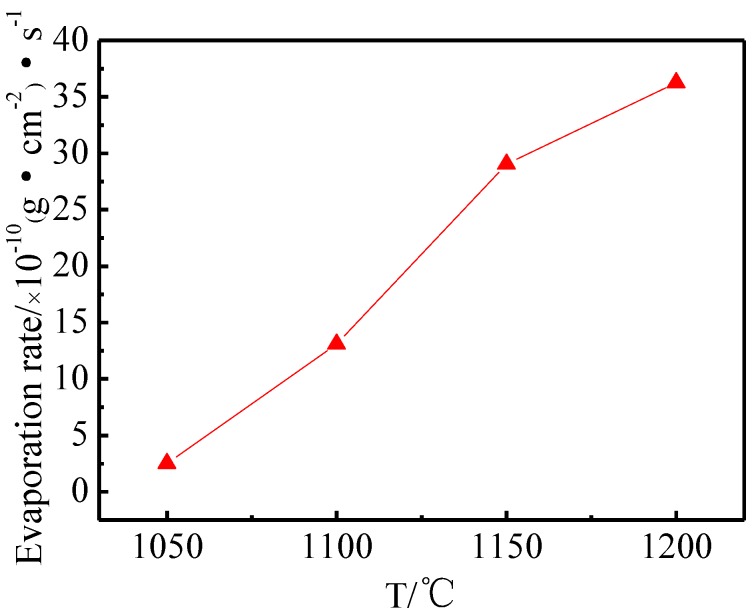
Average evaporation rate of cathodes filled with 6:2.0:2 aluminates at different temperatures.

Figure 8 shows the evaporation rate of the Ba-W cathode prepared by 6BaO·2CaO·2Al_2_O_3_ aluminates at different temperatures. The evaporation rate of the cathode increased with the increase of working temperature [31]. This was because the high temperature could promote the reaction of aluminate with W and thus produced more electron emitters. Therefore, the working temperature should be as low as possible when the current density was satisfied.

## 4. Conclusions

Five kinds of 6BaO·xCaO·2Al_2_O_3_ (x = 0.8, 1.2, 1.6, 2, 2.2) aluminates were prepared by liquid co-precipitation. The phases of aluminates before and after melting and their hygroscopic phase, melting characteristics, environmental stability, evaporation, and emission were studied. The main results are as follows:

(1) Except for 6BaO·0.8CaO·2Al_2_O_3_, the aluminates are a mixed phase of Ba_5_CaAl_4_O_12_ and Ba_3_CaAl_2_O_7_. The increase of the molar amount of CaO has little effect on the phase composition of aluminates, but it can increase the interplanar spacing. The molten phase of aluminates gradually changed from Ba_5_CaAl_4_O_12_ to the mixture phase of Ba_5_CaAl_4_O_12_ and Ba_3_CaAl_2_O_7_ as the molar amount of CaO increased.
(2) With the increase of the molar amount of CaO, the initial melting temperature and the total melting temperature of 6BaO·xCaO·2Al_2_O_3_ aluminates increased first and then decreased. The environmental stability of aluminates can be improved by properly increasing the molar amount of CaO, and the lowest 48 h weight gain rate of 6BaO·2CaO·2Al_2_O_3_ aluminates of single-phase Ba_3_CaAl_2_O_7_ is 10.88%. However, when the molar amount of CaO is more than 2.2, the environmental stability of aluminates becomes worse and the hygroscopicity increases due to lattice distortion that is caused by Ca^2+^. After exposure for 48 h, the diffraction peaks of aluminates are broadened to indicate the presence of Ca(OH)_2_, Ba_7_Al_2_O_10_, and unknown phases.
(3) The emission properties of aluminates can be improved with the increase of the molar amount of CaO. The maximum pulse emission current density of Ba-W cathode prepared by 6BaO·2CaO·2Al_2_O_3_ aluminates is 28.6 A/cm^2^ at 1050 °C However, when the molar amount of CaO exceeds 2.2, the emission performance of aluminates decreases. CaO can effectively reduce the evaporation rate of aluminate-Ba-W cathodes at working temperatures.

## Figures and Tables

**Table 1:** Properties of 6BaO·xCaO·2Al_2_O_3_ aluminates.

Mole Ratio of BaO:CaO:Al_2_O_3_	Phase Composition of Aluminates Prepared at 1500 °C for 2 h	Phase Composition of Aluminates Melted at 165 °C for 90 s	Initial Melting Temperature/°C	Total Melting Temperature/°C
6:0.8:2	Ba_3_CaAl_2_O_7_ Ba_5_CaAl_4_O_12_	Ba_5_CaAl_4_O_12_ Ba_3_CaAl_2_O_7_	1535	1546
6:1.2:2	Ba_3_CaAl_2_O_7_	Ba_3_CaAl_2_O_7_ Ba_5_CaAl_4_O_12_	1550	1560
6:1.6:2	Ba_3_CaAl_2_O_7_	Ba_3_CaAl_2_O_7_ Ba_5_CaAl_4_O_12_	1536	1546
6:2.0:2	Ba_3_CaAl_2_O_7_	Ba_3_CaAl_2_O_7_ Ba_5_CaAl_4_O_12_	1527	1547
6:2.2:2	Ba_3_CaAl_2_O_7_	Ba_3_CaAl_2_O_7_ Ba_5_CaAl_4_O_12_	1527	1537
